# Large-Scale 3–5 Hz Oscillation Constrains the Expression of Neocortical Fast Ripples in a Mouse Model of Mesial Temporal Lobe Epilepsy

**DOI:** 10.1523/ENEURO.0494-18.2019

**Published:** 2019-02-12

**Authors:** Laurent Sheybani, Pieter van Mierlo, Gwénaël Birot, Christoph M. Michel, Charles Quairiaux

**Affiliations:** 1 Functional Brain Mapping Laboratory, Department of Fundamental Neuroscience, University of Geneva, 1202 Geneva, Switzerland; 2 Neurology Clinic, Department of Clinical Neuroscience, University Hospital Geneva, 1206 Geneva, Switzerland; 3 Medical Image and Signal Processing Group, Ghent University, 9052 Ghent, Belgium; 4 Center for Biomedical Imaging (CIBM), 1015 Lausanne, Switzerland; 5 Center for Biomedical Imaging (CIBM), Geneva, Switzerland

**Keywords:** cross-frequency coupling, epilepsy, epileptic network, fast-ripples, oscillation

## Abstract

Large-scale slow oscillations allow the integration of neuronal activity across brain regions during sensory or cognitive processing. However, evidence that this form of coding also holds for pathological networks, such as for distributed networks in epileptic disorders, does not yet exist. Here, we show in a mouse model of unilateral hippocampal epilepsy that epileptic fast ripples generated in the neocortex distant from the primary focus occur during transient trains of interictal epileptic discharges. During these epileptic paroxysms, local phase-locking of neuronal firing and a phase–amplitude coupling of the epileptic discharges over a slow oscillation at 3–5 Hz are detected. Furthermore, the buildup of the slow oscillation begins in the bihippocampal network that includes the focus, which synchronizes and drives the activity across the large-scale epileptic network into the frontal cortex. This study provides the first functional description of the emergence of neocortical fast ripples in hippocampal epilepsy and shows that cross-frequency coupling might be a fundamental mechanism underlying the spreading of epileptic activity.

## Significance Statement

Focal epilepsies are no longer considered as diseases of a single brain region, but as a network disease. However, the mechanisms leading to the emergence of pathologic activity outside of the seizure-onset zone remain unknown. In this work, we show in a mouse model of temporal lobe epilepsy that large-scale slow oscillations of 3–5 Hz synchronize distant brain regions and constrain the expression of epileptic transient events in neocortical areas. This study establishes the first evidence that cross-frequency coupling promotes the expression of pathologic activity across an epileptic network, revealing that similar mechanisms operate to convey physiologic and pathologic activity across brain areas.

## Introduction

In physiologic conditions, long-range low-frequency activity supports the synchronization of distant brain regions involved in a joint task, while local processing occurs at higher-frequency bands (Von Stein and Sarnthein, 2000; Varela et al., 2001; Lakatos, 2005; Buzsáki, 2006; Womelsdorf et al., 2007; Fujisawa and Buzsáki, 2011; Lisman and Jensen, 2013). Evidence suggests that synchronization between brain areas aligns the excitability windows of neuronal populations, thus allowing for effective communication between the regions (Fries, 2005; Sadaghiani and Kleinschmidt, 2016). It is largely unknown whether these effective means of large-scale communication operate in epileptic brain networks.

Although the epileptic focus holds for the recurrence of epileptic seizures, focal epilepsies also involve large-scale interactions that are major pathogenic factors in epilepsy (Terry et al., 2012; Paz et al., 2013; Englot et al., 2016; Goodfellow et al., 2016; Smith and Schevon, 2016; Spencer et al., 2018). Poor prognosis is expected when pathologic activity emerges at a distance from the primary epileptic focus (EF): the existence of secondary generalized seizures (McIntosh, 2004) and bilateral interictal epileptic discharges (IEDs; Radhakrishnan et al., 1998) are associated with a lower rate of seizure freedom after the surgical resection of the EF. Furthermore, IEDs are also present at a distance from the focus or can travel across brain networks (Lantz et al., 2003; Vulliemoz et al., 2011), and these extrafocal activities impact cognitive functions in both human and animal models (Kleen et al., 2010; Ung et al., 2017). It is crucial to study the emergence of these remote pathologic activities and their dynamic interactions with the primary focus on understanding how epileptic activity can be elicited in distant brain regions, how a focal disease can lead to various debilitating symptoms (Hermann et al., 2008), and how we can prevent this (e.g., through responsive neuromodulation techniques; Zeitler and Tass, 2016).

How extrafocal interictal activities emerge in the epileptic brain is still unknown. We previously described in the kainate mouse model of hippocampal epilepsy that fast ripples (FRs) specific to the epileptic condition, while mostly present in the focus, are not confined to it and are also generated in the frontal cortices (Sheybani et al., 2018). To further explore the generation of these neocortical high-frequency oscillations, here we used multisite intracerebral recordings in both hippocampi and in the frontal cortex (FC) of awake head-fixed mice, previously injected with the proepileptogenic molecule kainate. Synchronization of brain regions before seizures has been shown to facilitate the spread of ictal activity (Khambhati et al., 2016), and low-frequency synchronization has been involved in the generation of interictal epileptiform activities (Matsumoto et al., 2013). Furthermore, pathologic high-frequency oscillations have been associated with local phase–amplitude coupling with a slow oscillation of 3–4 Hz, in contrast with physiologic high-frequency oscillations, which have been shown to be coupled with slower oscillations (0.5–1 Hz; Nonoda et al., 2016). This means that phase–amplitude coupling, although mainly studied in normal brain processing, might also be a fundamental property of pathologic brain activity. We hypothesize that slow oscillatory neuronal activity is related to the emergence of widespread pathologic activity in focal epilepsy and that cross-frequency coupling in the large-scale epileptic network might underlie the emergence of FRs in the frontal cortices.

We use time–frequency, phase–amplitude coupling and phase-locking analyses of local field potentials (LFPs) and multiunit activities (MUAs) around fast ripples to show that these epileptic events in the frontal cortex occur amid trains of IEDs and MUA bursts that are locked to a slow oscillation (3–5 Hz). Using phase analyses and partial directed coherence calculation across regions, we reveal that the frontal cortex and both hippocampi are synchronized around the frontal cortex FR within the 3–5 Hz frequency band.

We demonstrate a significant causality from hippocampal activity toward this slow oscillation, which eventually constrains the expression of the frontal IEDs. Altogether, our data identify a possibly fundamental property of how epileptic activity propagates and emerges in distant sites.

## Materials and Methods

### Animals and surgery

Eleven adult male C57BL/6J mice, including 7 kainate-injected and 4 saline-injected controls, were included in the study. Before kainate or saline injection, an aluminum ring-like header was attached to the mouse skull using dental cement. Surgeries were completed under isoflurane anesthesia (0.5–1%). A dot of ink was drawn on the skull above each region as a marker for the LH (left hippocampus) and RH (right hippocampus) regions [anteroposterior (AP), −2.67 mm; mediolateral (ML), ±2.5 mm] and FC (AP, 1.3; ML, 1.2) for recordings 4 weeks after injection. All experiments were conducted in accordance with Swiss laws on animal experimentation.

We worked on the widely used mouse model of hippocampal sclerosis, in which a hippocampal sclerosis with histopathological changes similar to human hippocampal sclerosis, associated with spontaneous epileptic seizures, is induced by unilateral intrahippocampal kainate injection (Arabadzisz et al., 2005). Kainate was injected under anesthesia (isoflurane, 0.5–1%) in the dorsal LH, as previously described [70 nl, 5 mm; Tocris Bioscience; LH: AP, −1.8; ML, 1.6; depth, 1.9 (using the Nanoinject, World Precision Instruments); Arabadzisz et al., 2005]. Control animals were injected with saline following the same procedure. The presence of hippocampal sclerosis was confirmed by Nissl staining.

### Awake electrophysiological recordings

Mice were trained to the head-fixed setup 1 week before the recording sessions started. A minimum of six training sessions (two daily) were necessary to obtain a sufficient animal compliance. Head-restrained recordings were achieved in the chronic stage of the disease (i.e., 4 weeks after kainate or saline injection; Arabadzisz et al., 2005), following similar procedures described in detail in the study by Sheybani et al. (2018). Three longitudinal A-16 Neuronexus electrodes were inserted into the LH and RH (AP, −2.67; ML ±2.5; depth, 2.02; angle, 20°) and the left FC (AP, 1.3; ML, 1.2; depth, 0.9; angle, 6°), and a reference electrode was placed on the posterior parietal bone under light anesthesia (isoflurane <0.5%). The choice of the frontal coordinates was driven by our previous findings of the emergence of neocortical FRs in this mouse model of hippocampal epilepsy (Sheybani et al., 2018). Once the electrodes were positioned, the anesthesia was stopped and the recording started after a minimal duration of 10 min to allow the animals to fully awaken (which usually occurred after 5 min). The daily recording session lasted 50 min. The signal was acquired at 16,000 Hz sampling rate using a Neuralynx acquisition system.

### Experimental design and statistical analysis

#### Detection of frontal cortex FRs using automated detector

The electrophysiological data were processed with Cartool [D. Brunet, Center for Biomedical Imaging (CIBM), University of Geneva, Geneva, Switzerland] and Matlab software (MathWorks). The identification of FRs was achieved using a detector based on criteria provided by the literature (Bragin et al., 1999; Lévesque et al., 2011). The detector filters (order 2 Butterworth filter) the data within the range of FRs (200–550 Hz) and then identifies events with at least four oscillations (i.e., four negative + four positive peaks) with amplitude more than three times higher than the SD of the ±250 ms surrounding baseline. All identified events were visually checked in the raw data to prevent false identification (e.g., artifacts of lower frequencies, such as epileptic spikes; Bénar et al., 2010). We then extracted a period of −200 to 600 ms around the onset of each frontal cortex FRs as the periods of interest (POIs; see Results) for further analyses. This period covers the duration of increased amplitude of the slow oscillation and of MUA phase-locking to the slow oscillation.

#### Construction of normalized time–frequency spectrogram centered around frontal FRs

We obtained the normalized time–frequency plots using the Stockwell transform (Stockwell et al., 1996) from 1 s windows locked on frontal cortex FRs. We obtained *z*-scored values by correcting with baseline following similar methods provided in the literature (Gelinas et al., 2016). The baseline windows were sampled randomly during periods of matching duration outside of seizures activity (determined as described in the study by Sheybani et al., 2018). The automatic randomized choice of baseline periods allows for nonbiased detection, although we cannot exclude that FRs randomly (and thus nonsystematically) occurred during these periods. A baseline spectrogram was first calculated for each animal. Then, for each trial within the same animal, a *z*-score was calculated using the same baseline mean and SD per frequency bin and time. To identify FRs associated with an IED, we selected events for which the maximal *z*-score was >2 within the frequency range of IED (i.e., 20–30 Hz). The slow oscillation onset for each FR was set at the time frame when the filtered signal went beyond 5 *z*-scores based on the median values of the time frames across each FR. A higher *z*-score threshold was chosen for calculation of the onset of the slow oscillation than for detection of the IED because we wanted a highly robust method to estimate the onset of the slow oscillation relative to the FR occurrence and to avoid attributing this onset to a nonspecific fluctuation.

#### Cross-frequency analyses

For all phase analyses throughout the study, data were first recalculated to bipolar montages (using the signal of two successive recording plots on the longitudinal A-16 neuronexus probes) to avoid any confounding effect of the reference. Analyses of phase-locking were achieved using the Rayleigh test for nonuniformity provided by the Matlab Circular Statistics Toolbox (Berens, 2009). MUA was detected according to the method of Quiroga et al. (2004), and phase-locking analysis was achieved using the instantaneous phase of the signal (by applying a Hilbert transform on filtered data). The method of Quiroga et al. (2004) allows for unsupervised MUA detection using a threshold detection defined as follows:

$$threshold=4*median\left(\frac{\left|x\right|}{0.6745}\right)$$

where *x* is the filtered signal (300–6000 Hz).

Phase synchronization was assessed during the POI (normalized over baseline) without any prior assumption on the frequency range (0–100 Hz). We calculated the difference of phases (obtained through a Hilbert transform of the filtered data) for each trial, and we calculated the parameter of concentration (κ) for each frequency bin (from 1 to 100 Hz; bandwidth, 3 Hz; step, 1 Hz). The parameter of concentration is high if the difference in phases is constant along the duration of one period of interest (all values concentrate toward a common value; i.e., there is synchrony). For statistical analysis, we applied the test for equal concentration parameter provided by the circular statistics toolbox (Berens, 2009) to test whether the synchronization within the identified frequency range was significant.

Cross-correlation matrices are obtained by translating two signals in the time domain and measuring the correlation at each degree of translation. A negative value indicates that the second signal is delayed in regard to the first.

Phase–amplitude coupling was obtained following the same procedure as described in the study by Tort et al. (2009). First, we calculated the amplitude and phase of the high-frequency (2–98 Hz; bandwidth, 3 Hz; step, 1 Hz) and low-frequency (2–20 Hz; bandwidth, 3 Hz; step, 0.5 Hz) components respectively obtained from the Hilbert transform of the filtered signal. Second, we binned the amplitude of the power (1–100 Hz) as a function of phase (1–20 Hz). Third, we calculated the mean amplitude per bin of phase for each FR. Fourth, we measured the Kullback–Leibler distance between the distribution of amplitudes along the phase and the uniform distribution, which assesses the degree of phase–amplitude coupling. Fifth, as in the study by Tort et al. (2009), we divided the values by a constant factor [i.e., log(18)], which gives the modulation index (MI) comprised between 0 and 1. This procedure was applied during the period of interest and control periods selected randomly in EEGs of the same animals for statistical purposes.

#### Integrated adaptive partial directed coherence to infer directionality of relationships among FC, LH, and RH

To determine directionality, we used the integrated adaptive partial directed coherence (iAPDC; Astolfi et al., 2008) a functional connectivity measure based on the concept of Granger causality (Granger, 1969). First, a time-varying autoregressive (TVAR) model is built from the multivariate time series using the Kalman filtering approach (Lie and van Mierlo, 2017). Afterward, the coefficients are transformed to the spectral domain using the Fourier transform to calculate the iAPDC.

$$iAPDC\left(t\right)=\frac{1}{f_{2}-f_{1}}\sum_{f=f_{1}}^{f_{2}}\frac{{\left|A_{ij}(f,t)\right|}^{2}}{\sum_{k=1}^{K}{\left|A_{ik}(f,t)\right|}^{2}}$$

with [*f_1_*, *f_2_*] meaning the considered frequency band, *K* the number of time series, and *A_ij_*(*f*,*t*) the Fourier transform of the TVAR coefficients that depicts the connection from time series *j* to time series *i* at time *t* and frequency *f*. The iAPDC*_ij_*(*t*) reveals the information flow from signal *j* to signal *i* in the predefined frequency band [*f_1_*, *f_2_*] at time *t*. The median iAPDC value was calculated from the intracranial recordings of the LH, RH, and FC during the 4 Hz oscillation and compared with that of baseline using the Kruskal–Wallis test. This was done to assess changes in directed connectivity among the LH, RH, and FC regions.

#### Analysis of phase consistency of the 3–5 Hz slow wave around FRs

Phase consistency across events was obtained using the phase-locking factor (Mazaheri and Jensen, 2006). To identify the phase across events with high temporal resolution , we used a wavelet transform. Each event was convolved with a complex Morlet wavelet using the same parameters as (Mazaheri and Jensen, 2006) and 199 frequency bins between 1 and 100 Hz. Then, for each event and each frequency bin, we obtained a control matrix by randomizing the phases along time. We thus obtained two matrices of three dimensions each (event × time frame × frequency bin). We then applied the test for equal concentration parameters (Circular Statistics Toolbox, Matlab; Berens, 2009) across events. The corrected alpha threshold corresponded to the usual 0.05 divided by the number of comparisons (number of time frames × number of frequency bins).

#### Statistical analysis

Prism (GraphPad Software) and Matlab were used for statistics and figures. Normality of data were assessed by the D’Agostino–Pearson test.

## Results

### A multiplex system: neocortical fast ripples occur amid transient slow oscillations and on top of interictal discharges

**Figure 1.**
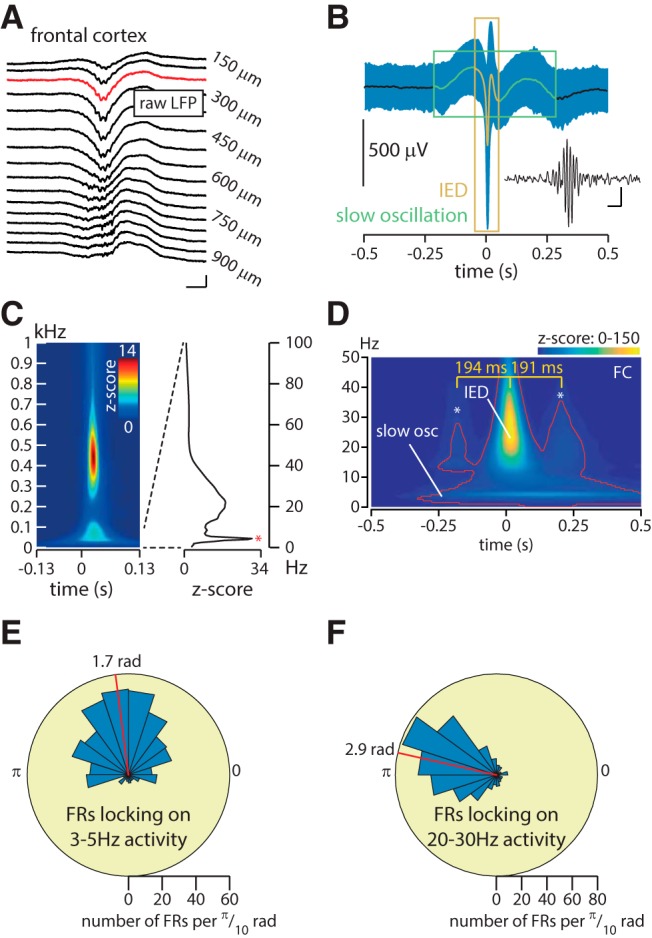
A multiplex system: neocortical fast ripples occur amid transient slow oscillations and on top of interictal epileptic discharges. ***A***, Example of a frontal cortex FR in raw LFP. Calibration: 800 μV, 10 ms. ***B***, Average raw LFP locked on FRs onset (mean ± SD; *n* = 729 in 7 kainate-injected animals). The green and orange rectangles highlight slow oscillations and IED components, respectively. The inset shows the FR in ***A***, filtered within 200–550 Hz. Calibration: 50 μV, 10 ms. ***C***, Average normalized time–frequency plot locked on frontal cortex FRs onset. Note the decline of spectral power separating the FR peak (∼450 Hz) from the LFP activity, indicating that frontal cortex FRs are not leaking artifacts of IEDs. Note that all frontal cortex FRs were visually checked in the raw data. Right, Average normalized (i.e., *z*-score) spectrogram from 0–100 Hz across all events at the FR onset showing the 3–5 Hz peak (red asterisk). ***D***, Time–frequency plot locked on frontal cortex FRs onset. Data included within the red contour are considered to be significant exceeding a threshold set at a *z*-score of 5 (a high *z*-score threshold is used given the large number of time–frequency points). The 0 time point indicates the frontal cortex FR onset. Frontal cortex FRs occur during transient trains of IEDs (***B***, orange), as shown by the increased power in the center of the window and the two peaks indicated by the stars. The duration between two successive peaks (194 and 191 ms) corresponds to the phase of an ∼5 Hz wave, suggesting that the IEDs are modulated by the slow oscillation (Fig. 3). Note also the increased power in the low frequencies (“slow osc”). Because frontal cortex FRs are time locked on IEDs, the central IED has a high *z*-score value, which overshadows the two surrounding IEDs and the slow oscillation, which are, however, significant (i.e., within the red contour). ***E***, ***F***, Circular histogram of the phase of the 3–5 Hz oscillation (***E***) and 20–30 Hz oscillation (***F***) at frontal FRs onset. The distribution is statistically nonuniform for both conditions (Rayleigh test for nonuniformity, *p* < 0.0001), confirming the existence of phase-locking. The red bar indicates the mean angle on which FRs are locked.

To further explore the generation of remote neocortical FR, we recorded neuronal activity in the FC, LH, and RH simultaneously in awake head-fixed animals 4 weeks after unilateral kainate (seven mice) or saline (four mice) injection into the left hippocampus. We identified 729 FRs (Fig. 1*A*,*B*) in the FC of kainate-injected animals compared with only 18 FRs in the saline-injected mice (median occurrence: 1.05/min in 7 kainate mice; 0.07/min in 4 saline mice; *p* = 0.0061, Mann–Whitney test). We first investigated whether these neocortical FRs, specific to the epileptic condition, occur in a “random” background, or whether they are associated with a specific ongoing activity. The mean LFP (1–100 Hz) locked on frontal cortex FRs (Fig. 1*B*) reveals two bandwidths associated with the FRs: a slow oscillation (3–5 Hz) surrounding the FRs (Fig. 1*B*, green box) and an associated IED (20–30 Hz; Fig. 1*B*, orange box). This IED range (20–30 Hz) is readily discriminated from the beta range on the time–frequency plot of Figure 1*D*. On single trials (Fig. 1*A*, negative deflection) and on the averaged activity (Fig. 1*B*), this 20–30 Hz peak can be visually identified.

**Figure 2.**
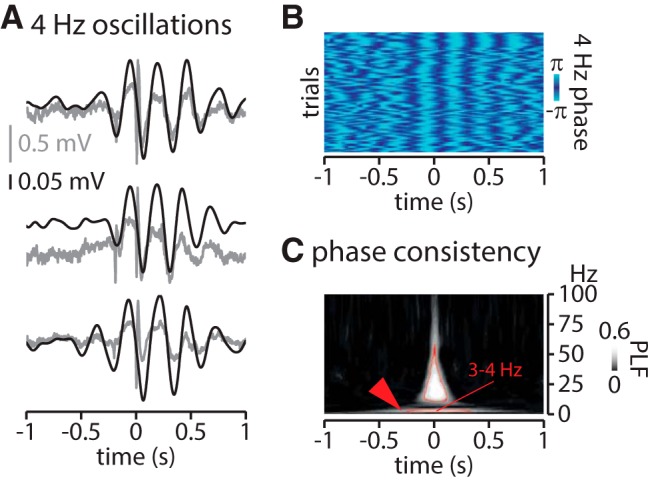
Local 3–5 Hz activity locked on frontal cortex FRs onset. ***A***, Three examples of 4 Hz oscillations around (± 1000 ms) frontal cortex FRs in the raw (gray) and filtered (3-5 Hz, black) LFP signal, in the FC. ***B***, Phase analysis across representative events (>200 events) showing the phase consistency around frontal cortex FRs (note the alignment of pale and dark blue around frontal cortex FRs onset). ***C***, Phase consistency across all events. Data within the red contour are significant (*p* < 6 × 10^−8^). PLF, Phase-locking factor as defined in the study by Mazaheri and Jensen (2006).

The observation of a slow oscillation at ∼3–5 Hz in the mean LFP activity can be due either to an increase in power or to a phase-locking of the frontal FRs (i.e., the markers for the analyses) around a slow oscillation. Time–frequency analysis around frontal cortex FRs, normalized by periods free of epileptic activity (see Materials and Methods), confirms the existence of an associated slow oscillation (Fig. 1*C*,*D*). Regarding the hypothesis of phase-locking, we indeed identify a significant locking of frontal FRs over the phase of the 3–5 Hz oscillation (Fig. 1*E*; Rayleigh test for nonuniformity, *p* < 0.0001). Furthermore, the slow oscillation can be easily identified on single trials (Fig. 2*A*), and aligning the phase values of LFP over 2 s windows centered over frontal cortex FR onset illustrates a phase consistency of the 3–5 Hz activity across events (i.e., FRs; Fig. 2*B*). The significant phase preservation index (Mazaheri and Jensen, 2006) confirms the alignment of the phase of the slow oscillation across events (Fig. 2*C*; see legends for details on statistical analyses per frequency bin). Thus, these analyses show both an increased power in the slow frequencies as well as an alignment of frontal cortex FRs over a specific phase of the slow frequency oscillations.

**Figure 3.**
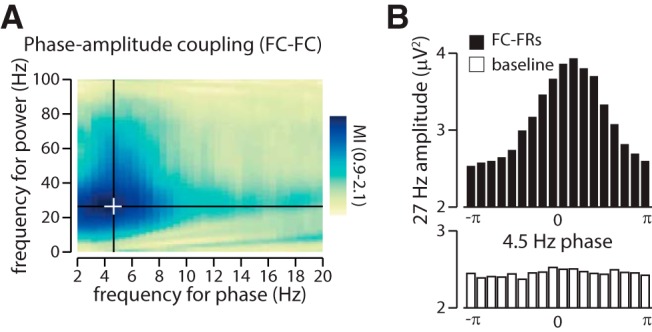
Phase–amplitude coupling ties IED amplitude to the slow-wave oscillation in the frontal cortex. ***A***, Phase–amplitude coupling (PAC) of the power of the IED (peak, 27 Hz) over the phase of the slow oscillation (peak, 4.5 Hz). The plot shows the ratio of the MI (period of interest divided by baseline). The MI is significantly higher during the period of interest than during the baseline (Mann–Whitney *U* test of the MI around frontal cortex FRs against MI during baseline, *p* < 0.0001), indicating that a slow wave constrains the expression of IED. The white cross indicates the peak in PAC. ***B***, Distribution of the 27 Hz power along the 4.5 Hz cycle (black, around frontal cortex FRs; white, baseline; i.e., at the peak of PAC; as identified by the white cross in ***A***).

As seen on Figure 1*B*, frontal FRs also seem to occur on top of IEDs, whose signature can be seen in the time–frequency analysis of Figure 1*D* at 20–30 Hz, and our analyses reveal that FR onset is also locked to the IED phase (Fig. 1*F*; locking on the 20–30 Hz activity; Rayleigh test of nonuniformity, *p* < 0.0001). Furthermore, the time–frequency analysis reveals two peaks in the IED frequency range (Fig. 1*D*, * symbol) that surround the central IED at delays suggesting a slow oscillation nesting of IEDs. To further investigate this potential nesting, we quantified the phase–amplitude coupling during these transient epileptic events (−200 to 600 ms around FC-FRs, “period of interest”) and during baseline activity. A significant phase–amplitude coupling was found during the period of interest between 4.5 Hz (frequency for phase) and 27 Hz (frequency for amplitude), which was 2.1 times higher than during baseline (Fig. 3*A*,*B*). The MI that assesses the modulation of the amplitude of an activity by the phase of another (Canolty et al., 2006) showed a significantly higher modulation for 4.5 Hz (for phase) and 27 Hz (for amplitude) during the period of interest compared with the baseline.

**Figure 4.**
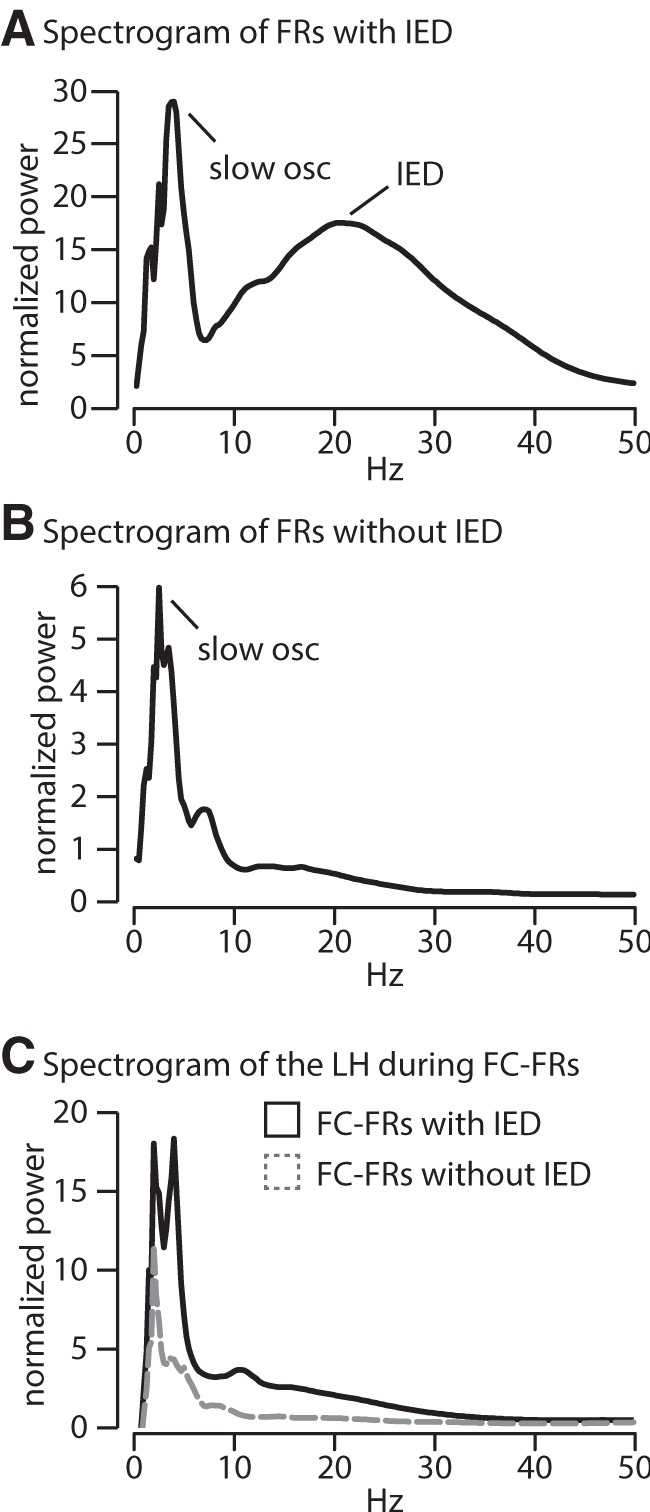
***A***, ***B***, Frontal cortex FRs can be associated, or not, with a local IED. Averaged spectrograms across FC-FRs associated (***A***, 481 events) or not associated (***B***, 248 events) with an IED. Both types of FC-FRs (i.e., associated or not associated with a local IED) show a significant normalized (*z*-score) activity within the slow oscillation frequency band, although the activity within this frequency band is lower for FC-FRs not associated with an IED. Only FC-FRs associated with an IED have the correspondent increase within the 20–30 Hz range, as expected. ***C***, Whether FC-FRs are associated or not with a local IED, they are not associated with the spectral signature (i.e., 20–30 Hz peak) of an IED in the LH.

Are all individual FRs associated with slow oscillations and IEDs? By *z*-thresholding the spectrograms (see Materials and Methods), we found that a total of 66% of FC-FRs are associated with a locally detectable IED. However, average normalized spectrograms show that FC-FRs are associated with a peak in the slow-wave power, whether they are associated with an IED or not (Fig. 4*A*,*B*). Altogether, these first analyses suggest a role of FR-IED and FR-slow oscillation phase-locking in the emergence of neocortical FRs. Furthermore, phase–amplitude coupling binds frontal IED amplitude to the slow oscillation phase. The interplay among different frequency bands that seems to be orchestrated by the slow oscillation calls for further investigation into the mechanism linking the slow oscillation to FC-FRs and into the source of this slow oscillation. In particular, is this frontal slow oscillation associated with activities in the epileptic network?

### The slow oscillation modulates neuronal firing over an ∼800 ms period around FRs

**Figure 5.**
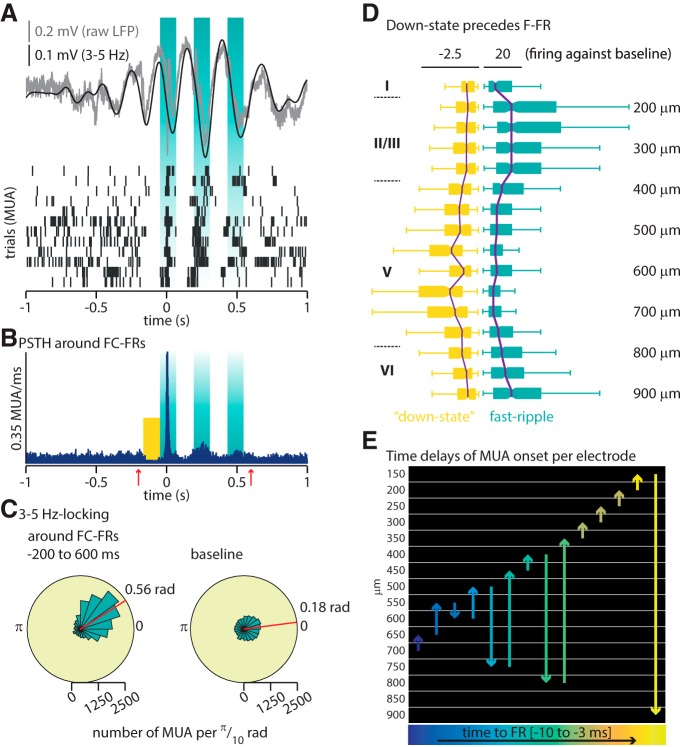
The slow oscillation modulates neuronal firing over an ∼800 ms period around fast ripples. ***A***, Top, Representative example of 3–5 Hz oscillation around one frontal cortex FR (not visible at this time scale) at time 0. Bottom, Twelve successive rasters of MUA around FRs during a representative recording showing the alignment of neuronal firing with 3–5 Hz oscillation. ***B***, Perievent time histogram of MUA around all identified frontal cortex FRs, illustrating that the MUA modulation is phase locked to the 3–5 Hz oscillation during an ∼800 ms period (onset and offset indicated by the red arrows, the so-called period of interest). The yellow square indicates the down state analyzed in ***D***. ***C***, Phase-locking of MUA over the 3–5 Hz slow oscillation phase. The phase-locking is significant for both the period of interest and the baseline (Rayleigh test, *p* < 0.0001 for both); however, the mean angle is significantly different [period of interest (0.56 rad) vs baseline (0.18 rad), *p* < 0.0001, Watson-Williams test for equal means], and the concentration parameter *κ* is higher during the period of interest (1.08 vs baseline at 0.32, *p* < 0.0001, test for equal concentration parameters). ***D***, Quantification of firing during the down state (yellow square in ***B***) and during FR (both normalized over the preceding baseline). Electrode depths are indicated on the right while roman numbers on the left label the approximate extent of cortical layers (agranular primary motor area). The scale corresponds to the ratio of MUA during the down state, and FRs divided by baseline; the center of each boxplot is the median, bars indicate the 25th and 75th percentiles (Kruskal–Wallis test, *p* < 0.0001). ***E***, Representation of the chronology of MUA onset across depth electrodes: time goes from left to right following the color code from blue to yellow. The *y*-axis corresponds to the depth of the neocortex in micrometers. Each arrow indicates the chronology of recruited layers (the length of the arrow has no meaning, and will vary only depending on the distance from one layer to another). As examples, the first arrow on the left (dark purple) indicates that the first layer with a significant increase of MUA is located at depth −700 μm, followed by layer located at −650 μm; the seventh arrow (green) indicates that the seventh layer with a significant increase of MUA is located at depth −450 μm, followed by layer located at −400 μm.

FRs are high-frequency activities (>200 Hz) resulting from the firing of a population of neurons (Foffani et al., 2007; Ibarz et al., 2010; Staba, 2012), whereas 3–5 Hz oscillations are much slower activities, raising the question of the dynamic link between these two different activities. Given the slow oscillation detected around frontal cortex FRs, we hypothesized that a possible mechanism could be a 3–5 Hz-based modulation of neuronal firing. We extracted 2 s periods centered on frontal cortex FRs and identified MUAs. As illustrated in Figure 5*A*, the MUA displays not only one transient increase locked on identified frontal cortex FRs but two to three bursts of increased neuronal firing separated by low firing bouts (Fig. 5*A*,*B*). These bursts (including the preceding down state) extend for ∼200 ms before the actual onset of FRs and up to ∼600 ms after, as seen on a perievent time histogram across all detected FC-FRs (*n* = 729; Fig. 5*B*). The dynamic of these bursts of MUA up and down states suggests they would align with a 3–5 Hz slow oscillation. In agreement with that, we confirm that the MUA is significantly phase-locked over the 3–5 Hz frequency during the POI (Fig. 5*B*, red arrows; i.e., from −200 to 600 ms around FRs; mean angle, 0.56 rad; SD, 1.02 rad; Fig. 5*C*). Although MUA is also locked to the 3–5 Hz oscillation during baseline (mean angle, 0.18 rad; SD, 1.3; Fig. 5*C*), the angle is significantly different between the POI and baseline, and the concentration parameter *κ*, which estimates the degree of locking, is higher during the POI than during baseline (Fig. 5, detailed statistics). Thus, neuronal activity is modulated by the slow oscillation during baseline and around FC-FRs, but the locking is stronger and translated toward a slightly different phase around FC-FRs (see Discussion).

Very low-frequency oscillations have been shown previously to associate with a cortical down state of neuronal activity in physiologic conditions, particularly in deep cortical layers (Steriade et al., 1993; Sirota and Buzsáki, 2005; Gelinas et al., 2016). We thus quantified neuronal firing across layers during the down state preceding the frontal cortex FRs (−180 to −30 ms) and during the frontal cortex FR (−30 to 20 ms). This revealed that the preceding down state is significantly more prominent in deep layers of the neocortex, whereas the increase of neuronal firing around FRs is significantly more prominent in superficial layers [Fig. 5*D*, Kruskal–Wallis test comparing the firing of each electrode during the down state (left, yellow) and during the FR (right, blue), *p* < 0.0001]. We then examined the onset of MUA per layer using time series of MUA (0, no action potential; 1, action potential) centered over frontal cortex FRs for each electrode (Fig. 5*E*). We convoluted this signal using a 10 ms Hanning window. Onsets of MUA activity were set when the amplitude of the convoluted signal between ±30 ms around the onset of FR reached three times the SD of the preceding baseline before the onset of the FR. Interestingly, the increase in MUA started slightly before the actual detection of frontal cortex FRs, and the onset was located initially in the deep layers of the FC and then propagated to more superficial layers (Fig. 5*E*). Given that MUA starts before FRs, both activities are not locked on the same phase of the 3–5 Hz oscillation (Fig. 1*E*, 1.57 for FRs; Fig. 5*C*, 0.56 for MUA), and consecutively the difference in mean angle (1.14 rad of a 35 Hz oscillation = 13.8 ms) closely approximates the difference in timing (10.1 ms).

Thus, frontal cortex FRs occur during short periods (∼800 ms) of increased neuronal firing, significantly modulated by a slow oscillation of 3–5 Hz. As the disease is initially induced in the LH, we next asked whether the hippocampi also express such a slow oscillation around frontal cortex FRs.

### A slow oscillation also occurs in both hippocampi around frontal cortex FRs

**Figure 6.**
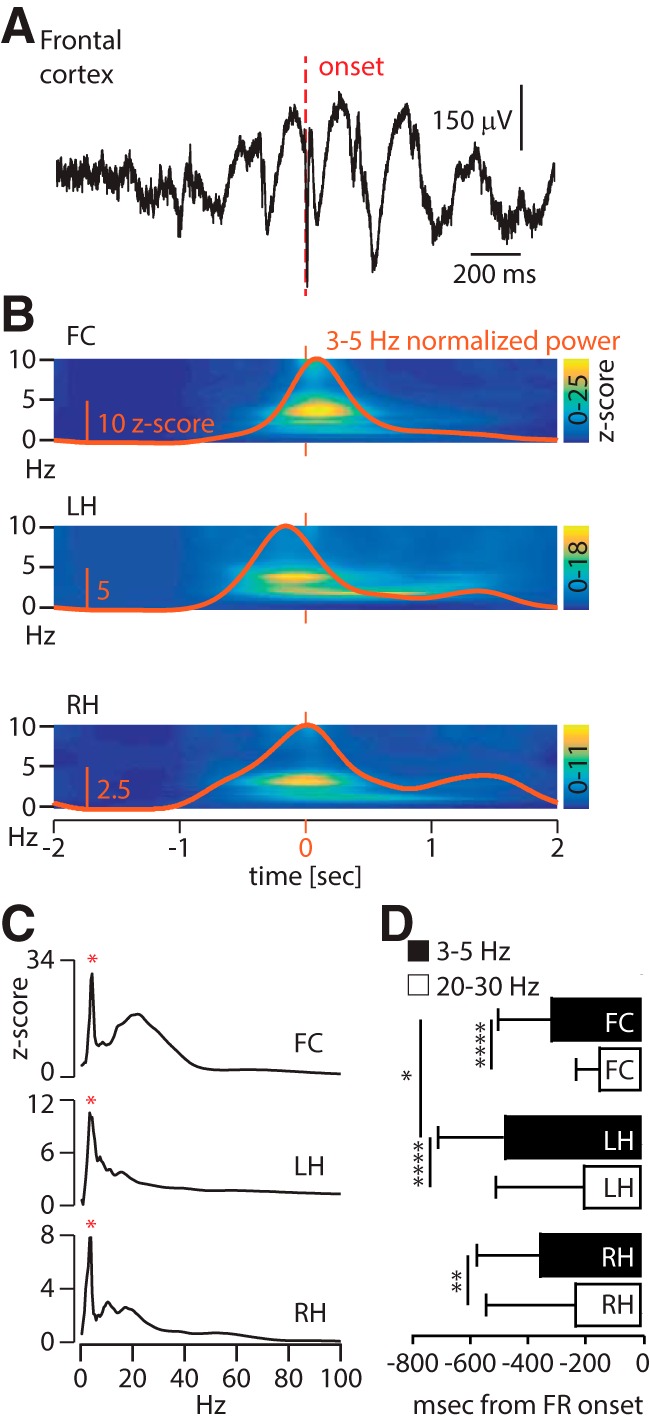
A slow oscillation also occurs in both hippocampi around frontal cortex FRs. ***A***, Raw signal around a typical frontal cortex fast ripple (not visible at this time scale) over a 2 s window, putting into light the slow oscillation on a single trial. ***B***, Population time–frequency plots (0–10 Hz, *z*-score) in the FC (top), LH (middle), and RH (bottom) around frontal FRs, illustrating the increase in power for the low frequencies (i.e., 3–5 Hz). The orange trace represents the values for the 3–5 Hz band only. The orange bars indicate the onset of FC-FRs (i.e., 0 ms). ***C***, Averaged spectrogram in the FC (top), LH (middle), and RH (bottom) normalized over baseline during the period of interest. Spectrograms show the signature of the slow oscillation in all three regions (red asterisk). ***D***, Median (± interquartile range) onset of 3–5 Hz activity in black and 20–30 Hz in white. The onset in the LH (−490 ms) precedes significantly the activity in the FC (−325 ms), while there is only a nonsignificant trend between the RH (−365 ms) and the FC; within each region, the onset of the slow oscillation anticipates the 20–30 Hz activity (Friedman test + Dunn’s multiple comparison test, *p < 0.05, ** *p* = 0.01, ****p < 0.0001).

We extracted windows of 4 s centered over frontal cortex FRs (Fig. 6*A*, period of interest) in the FC, LH, and RH. During this period of interest, the power specifically increases at ∼3–5 Hz in all three regions (Fig. 6*B*,*C*). We obtained averaged *z*-scored matrices of time–frequency analyses in these three regions (Fig. 6*B*) and observed a parallel and preceding increase in the low frequencies in both hippocampi centered on 3–5 Hz. To assess which of the three regions presents first with an increase in the 3–5 Hz activity, we identified the first time point at which the frequency activity reaches a significant level (set at a *z*-score of 5; see Materials and Methods). Using a paired analysis, we confirmed that the slow oscillation in the primary focus (i.e., the LH) started significantly before the one in the FC (−490 vs −325 ms), while there was only a nonsignificant trend when comparing to the RH onset (−365 ms; Fig. 6*D*). Only events for which the 3–5 Hz activity reached an increase in *z*-score of at least 5 in all three regions (LH, RH, and FC) were used to achieve the paired analysis.

Thus, FC-FR values are constrained by local slow oscillations, but these are concomitant with slow oscillations in the hippocampal network with the earliest onset in the hippocampal focus. These observations suggest that hippocampal slow oscillations might be a triggering event that anticipate and generate slow oscillations and FRs in the frontal cortex. Along with hippocampal slow oscillations, would it be possible that IEDs or FRs are generated in the focus before frontal FRs and that these events propagate to the frontal cortex to generate FC-FRs?

While 66% of the FC-FRs are associated with a local IED, only 22.5% (164 FC-FRs) are associated with an IED in the hippocampal focus. We measured the onset of IEDs in all three regions (as assessed by the onset of the 20–30 Hz activity) and found that there was a trend only in the onset of hippocampal IEDs to occur before FC-IEDs. The hippocampal IEDs occurred 55–85 ms before the frontal IEDs, but this difference was not significant (Friedman test with Dunn’s multiple-comparisons test; Fig. 6*D*). The onset of the slow activity, however, occurred significantly before the IED in all regions (Friedman test with Dunn’s multiple-comparisons test: LH and FC, *p* < 0.0001; RH, *p* < 0.001; Fig. 6*D*), and, as shown above, FC-FRs were associated with a peak in the slow-wave power whether an IED was present or not in the focus (Fig. 4*A*,*B*). Thus, these analyses are not in favor of IEDs as the major propagating events triggering FRs in the frontal cortex.

Then, we asked whether the FRs themselves could propagate across the network. If true, there should be a systematic detection of FRs in the hippocampi before their occurrence in the frontal cortex. On average, we found that FC-FRs are preceded by an FR in the LH and the RH in only 7% and 4% of the cases, respectively, during the 10 ms preceding each FC-FR, arguing against propagation. During a larger time window of 500 ms preceding the FC-FRs, we found an LH-FR in 62% of the cases, which was significant when compared with a baseline period (−1000 to −500 ms before FC-FRs; Wilcoxon test: LH, 62% vs 27%, *p* = 0.03) and an RH-FR in 10% of the cases (not significantly different from baseline). This suggests that while there is probably no propagation of FR, there might be a proexcitatory state of the network including the primary EF, leading to the co-occurrence of FRs across the network.

### A large-scale 3–5 Hz network originating from both hippocampi

**Figure 7.**
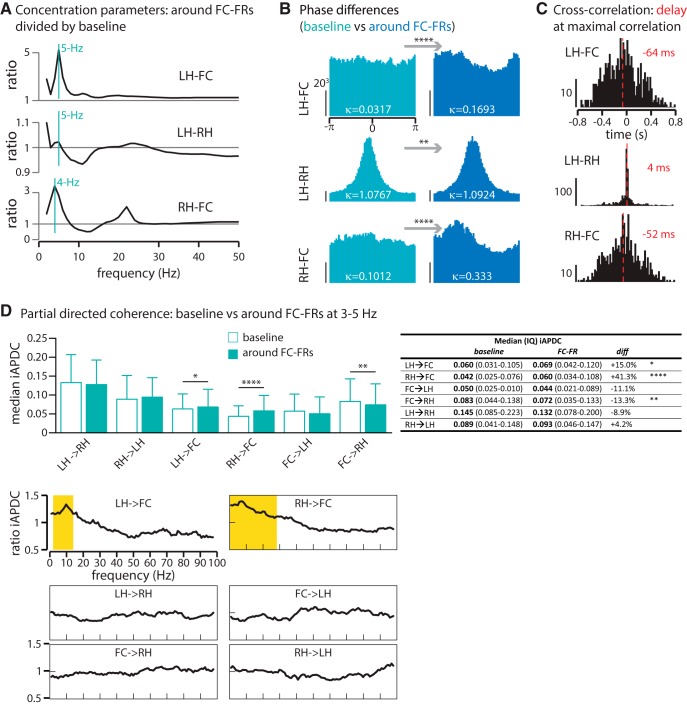
A large-scale 3–5 Hz network originating from both hippocampi. ***A***, Ratio of concentration parameters during the period of interest divided by baseline, along the LFP range. The 1 lines indicate no difference between frontal cortex FRs and baseline. In all three regions, but mainly for the couples LH-FC and RH-FC, the peak of synchrony is at 4–5 Hz. ***B***, Distribution of phase differences at each respective peak of synchrony (LH-FC, 5 Hz; LH-RH, 5 Hz; RH-FC, 4 Hz). For all three regions, phase differences are significantly more concentrated, indicating a synchronization across regions during the period of interest (test for equal concentration parameter κ, **p < 0.01, ****p < 0.0001). *Y*-scale, number of tested time frames per phase bin (for each subplot of ***B***, the vertical *y*-scale indicates 20^3^ tested time frames). ***C***, Cross-correlation at 3–5 Hz. The best correlation between activities in LH, FC, and RH is when the activity in the LH and RH is shifted backward by 64 and 52 ms, respectively, indicating that the LH and RH activity precedes the FC activity. When using the cross-correlation matrix between the LH and RH as a control (their delay being close to zero), we obtained a significantly different delay for the couples LH-FC and RH-FC (Kruskal–Wallis test, both at *p* < 0.0001). The *y*-axis indicates how many events (i.e., FC-FRs) present a maximal correlation at 3–5 Hz for each time-bin (*x*-axis). ***D***, Partial directed coherence (PDC) at 3–5 Hz (top). As shown, the driving from the LH and RH toward the FC increases significantly during the period of interest (Kruskal–Wallis test + Dunn’s *post hoc* test: *p < 0.05, **p < 0.01, ****p < 0.0001), supporting a causative role of both hippocampi in the 3–5 Hz frontal activity. Along the LFP range (below), the increased PDC is specific for low frequencies (LH toward FC, 2–14 Hz; RH toward FC, 0–29 Hz; Kruskal–Wallis test + Dunn’s *post hoc*, *p* < 0.0001, significant values in yellow). The table on the right compares the median iAPDC value for each connectivity measured during baseline and during FC-FR. The fourth column (diff) indicates the increase or the decrease in the iAPDC between baseline and FC-FR in percentages.

The parallel increase in the 3–5 Hz power range in all three regions suggests an increase in synchronization across regions. We first assessed this increase in synchronization by measuring the consistency of phase differences between regions (see Materials and Methods). When two regions synchronize their activity at a given frequency, the distribution of their phase differences should converge toward a common value. This convergence was quantified with the “concentration parameter.” Figure 7*A* shows the ratio of concentration parameters during the period of interest divided by the baseline period as a function of the frequency, revealing an increase in phase synchronization across brain regions within the 3–5 Hz frequency range (4 Hz for RH-FC and 5 Hz for LH-FC and LH-RH; Fig. 7*A*). The distributions of phase differences were significantly more concentrated during the period of interest than during baseline at these specific frequencies (Fig. 7*B*), demonstrating that the increase in the power of the slow oscillation is associated with the strongest synchronization between regions.

Second, we estimated the cross-correlation between regions within this specific frequency range (3–5 Hz). There was a significant delay of the FC activity over the LH and RH (64 and 52 ms, respectively; Fig. 7*C*), indicating that the activity of both hippocampi precedes the one in the FC. While cross-correlations indicate that modifications of activity first occur in the LH and RH, and then in the FC, it gives no definitive measure of causality and directionality between these activities. We therefore used Granger algorithms as a measure of causality prediction to assess this. We measured the Granger causality during baseline and during the period of interest, between all pairs of three regions, at the 3–5 Hz frequency range. By comparing these indices of causality, our results suggest that driving from both hippocampi toward the FC is significantly stronger around the FC-FR compared with baseline, while driving from the FC to the hippocampus decreases. This driving activity was specific for low frequencies and for both hippocampi toward the FC, although the driving frequencies extended somewhat to higher frequencies (Fig. 7*D*, bottom).

Altogether, these data reveal that while cross-frequency coupling between the slow oscillation and MUA constrains the expression of FC-FRs locally, this slow oscillation arises in the bihippocampal network then spreads across the network before FC-FRs expression. Granger causality analyses suggest that these oscillations exert a significant driving from both hippocampi toward the frontal cortex.

## Discussion

This study provides the first functional description of the emergence of neocortical FRs in hippocampal epilepsy, and establishes cross-frequency coupling as a fundamental mechanism underlying the spreading of epileptic activity. Using multiple-site recordings in awake animals previously injected with saline or the proepileptogenic molecule kainate, we first show that neocortical FRs in hippocampal epilepsy are not merely isolated events, but occur within complex epileptic transients characterized locally by a cross-frequency coupling between a slow oscillation and bursts of IEDs, and a strong and focal modulation of neuronal firing by the slow oscillation during ∼800 ms. We hypothesized that the link between the very low-frequency and very high-frequency activities might be a slow oscillation-based modulation of neuronal activity, as follows: the slow oscillation, by concentrating the MUA at a specific phase, might increase the likelihood to trigger FRs, which are known to be generated by bursts of population spikes (Staba, 2012). We observed that MUA is phase-locked during baseline and the POI, indicating that neuronal activity is modulated by the slow oscillation in both conditions. The fact that the locking is more precise and slightly but significantly translated toward a different phase during the POI suggests that, while the slow oscillation is probably necessary, it is, however, not sufficient to set neurons to express FRs. Therefore, other mechanisms remain to be elucidated. It might be that a pathologic neuronal assembly, and not all neurons, changes its coupling with the slow oscillation; clustering analyses should help to discriminate between neurons that modify their locking to promote FR expression and those that do not. One interpretation could be that during the course of the disease, abnormal neuronal networks are formed and consolidate in brain regions beyond the focus that become part of a large-scale epileptic network. The choice of 3–5 Hz range for the slow oscillation is data driven (i.e., based on the peak of the slow oscillation identified in the time–frequency plots; Fig. 1*D*), and further correspond to (1) the peak of phase consistency (Fig. 2*C*), (2) the peak of phase–amplitude coupling in Figure 3*A*, (3) the peak of spectral analysis in Figure 6*C*, and (4) the peak of synchronization between regions (LH, RH, and FC) depicted in Figure 7*A*. Our approach, which is not based on the classical delta to high-gamma segregation of frequencies favors a nonarbitrary selection of the specific frequencies involved in the mechanisms discussed in the project.

Extending our analyses to the network level, we demonstrate that both hippocampi also express a slow oscillation during these events. Finally, we show that the bihippocampal slow activity precedes and ultimately predicts the slow oscillation in the FC. Altogether, our data uncover a fundamental role of cross-frequency coupling in the emergence of remote epileptic discharges in focal epilepsy. High-density, surface EEG suggested that the motor region of the frontal cortex is a major region for remote FR expression (Sheybani et al., 2018), although FRs were also detected in other brain areas, and therefore this region is one of the potential targets for the epileptic pathways in this model. Furthermore, given the delays of the activities and cross-correlations between regions and in the absence of known (until now) direct projections from the hippocampus to the frontal motor cortex, one should hypothesize that this pathway is polysynaptic.

A central question to this work is to understand what activity (i.e., the slow activity, the IEDs, or the FRs) is driving the others. Based on the timing of slow oscillations in all three regions (Fig. 6*D*), the cross-frequency analysis (Fig. 7*C*) and the Granger causality analysis (Fig. 7*D*), it is presumable that the slow oscillation is the propagating event in the network. In other words, the hippocampal slow oscillations generate output activities driving frontal slow oscillations that in turn would modulate local neuronal firing and favor the generation of FRs in the frontal cortex of the epileptic brain. On the contrary, IEDs themselves are not well suited to be the major propagating events because (1) the onsets of IEDs are not significantly different between regions, (2) IEDs are preceded by the slow oscillations in all regions, and (3) because IEDs are detected in the epileptic focus for only 22.5% of the FC-FRs. This subfraction of the FC-FRs concomitant with a hippocampal IED might correspond to the generalized spikes observed in surface EEG during which IEDs propagate to all brain regions (Sheybani et al., 2018). Finally, we do not believe that FRs are themselves propagating because FRs are detected in close proximity (10 ms) with the FC-FR in only 7% of the case in the focus and in 4% in the contralateral hippocampus. Rather, we interpret the significant increase of FRs in the focus during the 500 ms preceding FC-FRs as a signature of a proexcitable network.

Slow waves are frequently observed after IEDs, corresponding to a depression of neuronal activity (Noebels and Jasper, 2012). We believe that a comparable mechanism may take place during the transient oscillations in the frontal cortex, yet the earlier onset of the slow activity (compared with the IED onset) renders the IED as the triggering event less likely. We believe that the slow oscillation concentrates neuronal activity during a specific phase (as illustrated by the phase locking in Fig. 5*C*), eventually leading to an IED. Then, the following wave most probably obeys the same mechanisms as the slow component of IEDs (Alarcón et al., 2012). To note, the finding that some cells decrease their activity during IED, without a previous discharge (Alarcón et al., 2012), could suggest that the slow wave is already the expression of a dysfunctional network, and not only the IED.

In physiologic conditions, cross-frequency coupling represents a strong candidate for effective communication between neuronal populations (Fries, 2005; Sadaghiani and Kleinschmidt, 2016). Ongoing oscillations open and align transient windows of increased excitability of neuronal populations, thus fostering the response of a neuron to a given input (Buzsáki and Draguhn, 2004). In this context, a pioneering work showed in the rat that a 4 Hz oscillation synchronizes distant brain regions within a large-scale network dedicated to working memory during an odor–place matching task (Fujisawa and Buzsáki, 2011). This oscillation constrains neuronal firing, and this modulation is enhanced for goal-predicting neurons (i.e., those neurons that increase their firing significantly, depending on whether the animal will go on the left or right side of the maze; Fujisawa and Buzsáki, 2011). Higher-frequency activity seems to coordinate more local circuits, such as hippocampal–entorhinal circuits in memory formation, which was shown to rely on 20–40 Hz coherence (Igarashi et al., 2014). While these efficient means of neuronal communication have been demonstrated in physiologic processing, their involvement in the emergence of epileptic activity in distant sites is still largely unknown. Facilitation of IEDs and high-frequency oscillations has been shown during slow oscillation (Frauscher et al., 2015), and, at least in the EF, Nonoda et al. (2016) showed a coupling between the amplitude of high-frequency oscillation and the phase of a 3–4 Hz oscillation. Thus, evidence suggests an association between oscillations and epileptic transients, such as IEDs or high-frequency oscillations. However, a precise description of the mechanism is still missing; here, we add new evidence of a causative property of slow oscillation from one brain region to another, and their involvement in tuning neuronal firing and organizing IED expression in areas remote from the focus. The observation that a 4 Hz oscillation is observed across a large-scale epileptic network in our work and across a physiologic one (Fujisawa and Buzsáki, 2011) is intriguing and might reflect the possibility that this range of oscillation subserves communication between brain areas, whether pathologic or not.

Interictal epileptic activities are essential epileptic transients to study, as they are known to be associated with symptomatic deficits in animals (Aldenkamp and Arends, 2004; Kleen et al., 2010; Gelinas et al., 2016) as well as in humans (Aarts et al., 1984). Intracerebral and surface EEG reports have shown that FRs can be elicited at distance of the focus and in nonepileptic patients, and intense research aims at discriminating between physiologic and pathologic FRs (Frauscher et al., 2018; Mooij et al., 2017). However, FRs, as well as IEDs, can be used as indicators of epileptogenic brains regions (Jacobs et al., 2010; Mégevand et al., 2013) and were shown to predict surgical outcome (Wu et al., 2010; van’t Klooster et al., 2015). The powerful advantage of using FRs as a proxy for epileptic activity is their remarkable standardized pattern (see their definition in Materials and Methods), which allows decreasing the variability peculiar to IEDs. In a rat model of hippocampal epilepsy, Gelinas et al. (2016) demonstrated a correlation between hippocampal IEDs on one hand, and spindles in the medial prefrontal cortex on the other. Interestingly, the association between IEDs and spindles correlates with poor memory performance. Immediately after the IED, they identified a positive delta wave associated with a deep-layer down state in the medial prefrontal cortex (Gelinas et al., 2016). However, Gelinas et al. (2016) investigated how a pathologic activity associates with a physiologic one (i.e., prefrontal spindles), and not the emergence of remote pathologic activity. Concerning the possibility that neuronal firing might be modulated by slower activity, this was already shown, yet only in the primary focus and in the direct vicinity of IEDs (Keller et al., 2010; Matsumoto et al., 2013) i.e., not during several cycles of an oscillatory activity. Thus, current knowledge still lacks the mechanistic insight supporting the emergence of pathologic activity in extended brain networks. We were first interested in the identification of remote pathologic activities and their local generation, and then in the involvement of the bihippocampal circuit. The hypothesis of a slow-oscillation involvement in the expression of large-scale epileptic activities was confirmed by several different tests, including phase difference analysis, Granger causality measures, and MUA phase-locking.

Together, our data support cross-frequency coupling as a basic mechanism for the propagation and the expression of epileptic activity in the large-scale epileptic network, beyond the focus. This allows us to speculate that such mechanisms of synchronization might be important for maintaining synaptic weights in the epileptic network and that manipulation protocols, such as responsive neurostimulation using closed-loop devices, or resetting protocols of neuronal activity (Zeitler and Tass, 2016), may be valuable therapeutic options in the future. A strategy could be to use large-scale recordings to identify proexcitatory windows, which are coordinated and hence predictable by the slow oscillation, as targets of brain stimulation therapies.

## Synthesis

Reviewing Editor: William Stacey, University of Michigan Decisions are customarily a result of the Reviewing Editor and the peer reviewers coming together and discussing their recommendations until a consensus is reached. When revisions are invited, a fact-based synthesis statement explaining their decision and outlining what is needed to prepare a revision will be listed below. Note: If this manuscript was transferred from JNeurosci and a decision was made to accept the manuscript without peer review, a brief statement to this effect will instead be what is listed below.

The reviewers and I have discussed the manuscript. There have been significant improvements, and we would like to publish the paper in eNeuro, provided there are some minor changes. We would like to have a minor revision (editorial review only) to address a few points of clarification.

1. Add some statements that this particular region in the frontal lobe is just one of many potential pathways. You might also want to discuss that the delays between Hipp and FC may suggest polysynaptic connections, though this is not mandatory.

2. Please temper the statements about “driving” and causality, as these have not been indisputably proven. Examples are in the paragraph lines 453-464, which implies that that Granger causality has demonstrated that the hippocampus is driving the FC. This is not necessarily true. Using language such as that the Granger results were “predictive of causality” is preferred.

3. Explain how the IED range (20-30 Hz) is discriminated from normal physiological activity in the beta range? Also please comment on the choice of 3-5 Hz, as it contains both delta and theta--does this have any potential significance for the functional boundaries of these bandwidths?

4) Some phrasing should be corrected, as the word usage is ambiguous. Line 82, “Dreaded complications” is very vague. Line 121 - “propagation and emergence in distant site” - Emergence of what? Line 567 - entanglement = involvement?

5) In Fig. 5D, there should be a general description of which cortical layers are expected to be in each depth.

6) In 5E, the length and direction of the arrows should be explained. This figure does not make sense without better explanation.

7) In Fig. 7D, please clarify what it means that the FC-RH relationship is a negative number. The paper talks about directionality and causality, so displaying a negative number for FC-RH and a positive one for RH-FC needs to be explained better in this context.
